# Mechanics of Psoas Tendon Snapping. A Virtual Population Study

**DOI:** 10.3389/fbioe.2020.00264

**Published:** 2020-03-27

**Authors:** Emmanuel A. Audenaert, Vikas Khanduja, Peter Claes, Ajay Malviya, Gunther Steenackers

**Affiliations:** ^1^Department of Orthopedic Surgery and Traumatology, Ghent University Hospital, Ghent, Belgium; ^2^Department of Trauma and Orthopedics, Addenbrooke’s Hospital, Cambridge University Hospitals National Health Service Foundation Trust, Cambridge, United Kingdom; ^3^Op3Mech Research Group, Department of Electromechanics, University of Antwerp, Antwerp, Belgium; ^4^Department of Human Structure and Repair, Ghent University, Ghent, Belgium; ^5^Medical Imaging Research Center (MIRC), University Hospitals Leuven, Leuven, Belgium; ^6^Department of Electrical Engineering/Processing Speech and Images, KU Leuven, Leuven, Belgium; ^7^Department of Human Genetics, KU Leuven, Leuven, Belgium; ^8^Murdoch Children’s Research Institute, Royal Children’s Hospital, Melbourne, VIC, Australia; ^9^Department of Orthopedic Surgery and Traumatology, Northumbria National Health Service Foundation Trust, Newcastle upon Tyne, United Kingdom; ^10^Department of Regenerative Medicine, Institute of Cellular Medicine, Newcastle University, Newcastle upon Tyne, United Kingdom

**Keywords:** tendon mechanics, ischiofemoral impingement, muscle wrapping, geometric morphoinetric analysis, virtual population

## Abstract

Internal snapping of the psoas tendon is a frequently reported condition, especially in young adolescents involved in sports. It is defined as an increased tendon excursion over bony or soft tissue prominence causing local irritation and inflammation of the tendon leading to groin pain and often is accompanied by an audible snap. Due to the lack of detailed dynamic visualization means, the exact mechanism of the condition remains poorly understood and different theories have been postulated related to the etiology and its location about the hip. In the present study we simulated psoas tendon behavior in a virtual population of 40,000 anatomies and compared tendon movement during combined abduction, flexion and external rotation and back to neutral extension and adduction. At risk phenotyopes for tendon snapping were defined as the morphologies presenting with excess tendon movement. There were little differences in tendon movement between the male and female models. In both populations, abnormal tendon excursion correlated with changes in mainly the femoral anatomy (male *r* = 0.72, *p* < 0.001, female *r* = 0.66, *p* < 0.001): increased anteversion and valgus as well as a decreasing femoral offset and ischiofemoral distance. The observed combination of shape components correlating with excess tendon movement in essence presented with a medial positioning of the minor trochanter. This finding suggest that psoas snapping and ischiofemoral impingement are possibly two presentations of a similar underlying rotational dysplasia of the femur.

## Introduction

Internal snapping hip, also referred to as coxa saltans or “dancer’s hip,” is a relatively common and potentially debilitating disorder produced by the iliopsoas tendon slipping over soft tissue and bony prominences during hip movement, causing an audible clicking that is typically accompanied by pain. The condition was originally described by Nunziata and Blumenfeld (1951) and garnered growing interest in the last decade, coinciding with the increasing use of hip arthroscopy. The pioneering authors described the snapping as a consequence of the proximal portion of the iliopsoas tendon gliding over the iliopectineal eminence (Lyons and Peterson, 1984). Howse (1972), however, believed that a “clicking hip” in ballet dancers was caused by the iliofemoral ligaments sliding over the femoral head. These historical suppositions were mainly speculative, with little supporting scientific evidence.

Jacobson and Allen (1990) were among the first to perform anatomical and radiographic studies, associating the iliopsoas tendon snap with its movement across the femoral head from lateral to medial with the hip brought to extension. The exact pattern of psoas tendon movement, however, remains debated and unresolved to date, with most authors claiming snapping to occur either over the iliopectineal eminence or the anterior part of the caput femoris (Taher and Power, 2003; Verhelst et al., 2012; Lee et al., 2013; Philippon et al., 2014; Ilizaliturri et al., 2015; Yen et al., 2015). Furthermore, several anatomical variations and focal pathological conditions, e.g., labral cysts, have been reported to increase the likelihood of occasional snapping to become recurrent and therefore clinically symptomatic.

While the location of tendon movement remains debated, literature does agree on the hip positions which can provoke coxa saltans. In all descriptions of the condition it occurs predominantly when the hip is moved from combined flexion, abduction, and external rotation (FABER) back to neutral adduction and internal rotation (Winston et al., 2007; Yen et al., 2015). This explains why snapping hip can be provoked when climbing stairs or when one gets out of a car or gets up from a chair. This also explains its higher incidence in athletes who walk, dance, lift weight and play football (Lee et al., 2013). For competitive ballet dancers it is reported that symptoms mount up to 90% and bilateral involvement as high as 80% (Nolton and Ambegaonkar, 2018). Winston et al. (2007) reported snapping in ballet dancers to occur almost exclusively during abduction-external rotation activities: grande battement à la seconde (a high kick to the side with a straight leg) in 41.8%, grand plié (a deep knee bend) in 25.3%, and développé à la second (a slow extension of the leg from the FABER position) in 22.8%. In the general population the prevalence of audible snapping is estimated at 5–10%, with a higher frequency in females as compared with males (Taher and Power, 2003; Lee et al., 2013; Yen et al., 2015).

Despite the condition being relatively common, little is known about the exact mechanism or origin of psoas tendon snapping. Notwithstanding the numerous theories proposed on the topic, in depth analysis of psoas mechanics during movement has not been carried out previously. This is mainly due to its difficult dynamic visualization and the condition therefore largely remains unsubstantiated.

## Related Work

### Tendon-Muscle Path Prediction: Muscle Wrapping Algorithms

The shortest path problem of tendon-muscle path predictions is one of the oldest biomechanical challenge. The earliest techniques to represent and predict a tendon-muscle path adopted “via” points connecting straight line segments (Delp and Loan, 1995; Kruidhof and Pandy, 2006). Later authors improved upon this approach by the use of passive geometric constraints -e.g., cylinders, spheres, and ellipsoids- over which these line segments are wrapped (Arnold et al., 2000; Garner and Pandy, 2000; Charlton and Johnson, 2001; Audenaert and Audenaert, 2008). These so -called “obstacle-set” methods have been successfully applied in upper and lower limb models and resulting estimates for muscle location, lengths, and moments have been shown comparable to experimental measurements (Vasavada et al., 2008). Probably the most important property of this approximate method is speed of calculation, allowing for real time visualization of tendon-muscle paths during motion. In addition, these predictions can be used as “seeding” position for more complex volumetric wrapping techniques (Kohout et al., 2013) or full 3D finite element (FE) models of muscle (Reynolds et al., 2004; Blemker and Delp, 2005). In parallel with this evolution, research has focused on more anatomical descriptions of the 3D geometry of adjacent bony and soft tissue structures as limiting constraints, adding to the complexity of an already challenging problem (Gao et al., 2002; Marai et al., 2004; Desailly et al., 2010; Liu et al., 2012; Kohout et al., 2013; Hammer et al., 2019).

In spite of the obvious advancement in anatomical authenticity, these techniques have, however, been facing the challenges of increased complexity and associated computational requirements, restricting their usage in real time or population size applications as compared to the traditional obstacle-set methods. A valid and computational efficient alternative over personalized anatomical FE models, however, was recently proposed by Audenaert et al. (2019a) using rigid body spring models. This discrete element (DE) model provides an anatomical truthful description of tendon-muscle paths as they wrap over any anatomical, geometric or combined sets of surface structures, while allowing for fast processing of large simulations series (Audenaert et al., 2019a). The present work makes use of this recent technique to investigate the problem of psoas tendon movement in a population scale model.

### Articulated Shape Models and Virtual Population Models

While simulation of tendon-muscle positions using finite or discrete element analysis has been shown to be reliable on a patient specific basis, replication of such analyses across large populations introduces an additional ethical, logistical, and computational challenge in order to obtain the required data set (Audenaert et al., 2019b,c, 2020). In that respect, population-based modeling has been applied to meaningful effect in biomechanics by capturing the effect of anatomical variation in many body regions (Rao et al., 2013; Bischoff et al., 2014; Campbell and Petrella, 2016). The description of shape variation at the population level a challenge, necessitating considerable training samples to reach acceptable generalization properties of the models. The number of training samples required for covering population variance of anatomical structures is scarce in the literature. In the area of face recognition, it has been documented that in order to representatively achieve population coverage, at least 250 samples are required (Claes, 2007). Similar numbers of training samples have recently been described to be required in osteological models (Audenaert, 2019). In order to apply statistical shape modeling in the evaluation of joint mechanics, one must consider the multiple structures comprising the joint, their interdependencies and their relative alignment (Rao et al., 2013). Differences in pose among scanned subjects at the time of the image acquisition, however, adds undesirable intersubject variability that does not describe variance in patient anatomy. Because of this issue and for the purpose of modeling the lumbar spine, Rasoulian et al. (2013) previously argued that shape and pose should be deled with separately, as they are not correlated. Other authors have tried to handle the difference between alignment and pose either by defining statistical transformation models to capture differences in pose (Boisvert et al., 2008) or by explicitly modeling motion trough idealized joint models (e.g., a ball and socket joint with three degrees of freedom for the hip or hinged joint in case of the knee joint) (Kainmueller et al., 2008, 2009; Rao et al., 2013). We recently developed a novel method to decrease pose variance to increase compactness and thereby usability of multi-component shape models based on learning methodology and not enforcing idealized kinematic definitions on the joints (Audenaert et al., 2020). For the present study and with the aim to investigate the relationship between hip anatomy and psoas movement, the latter approach was implemented. In particular we hypothesized snapping would be associated with particular anatomical variation of pelvis and femur.

In summary, the objective of this manuscript is double. First, we propose to the best of our knowledge the first computational model of the lower limb to investigate psoas tendon behavior in a population sized approach. Secondly, we test the hypothesis that the variation in anatomy is correlated with abnormal tendon excursions during movement.

To this end, the following key technical challenges will be addressed: (1) We describe a representative virtual population of pelvifemoral models, within which psoas anatomy are derived. (2) We simulate psoas tendon behavior during provocative hip motions, which is a critical step in understanding why some anatomical configurations might be associated with abnormal psoas tendon excursion.

## Methods

### Patient Population and Kinematic Protocol

The Ghent Lower Limb model was used to generate a virtual cohort of 20,000 male and an equal number of 20,000 female cases, representative for a western European population. The model was previously validated in terms of population coverage and model specificity, generalizability and accuracy. It is composed following principal component analysis of dens corresponding meshes obtained from 544 lower limb segmentations (362 male and 182 female training samples) (Styner et al., 2003; Audenaert, 2014, 2019, Almeida et al., 2016; Audenaert et al., 2019c). After segmentation and registration of the anatomical shape entries, male and female shape entries were separated and used for the construction of two distinct PCA models to describe shape variance Mathematically, each model was described using the following equation:

$$S=\bar{S}+P\,b$$

where S is a vector of size 3n representing the shape in terms of n 3D landmark points. In the equation above $\bar{S}$ corresponds to the average shape of the model and P represents a t × 3n matrix encapsulating t eigenvectors describing the principal directions of variation of the model. Further, every unit vector is associated with an eigenvalue λ_*i*_, i ∈ {1, ⋯, t} which describes the magnitude of variation along each axis. Lastly, b = (b_1_, ⋯,b_*t*_) represents a vector containing the b_*i*_ weights that regulate the deviation of the shape S from the mean and follow a normal distribution.

**FIGURE 1:**
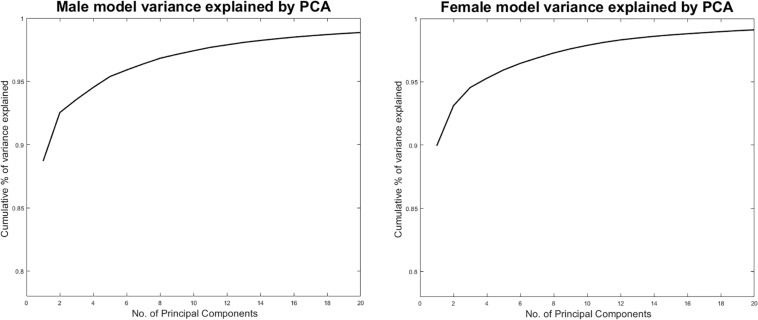
Cumulative male and female variance explained by PCA.

For both sexes, it was determined that 20 shape modes were necessary to describe a sizable 99% of anatomical variance (Figure 1). We therefore retained *t* = 20 modes of variation to generate the virtual shapes, by randomly varying the normal distributed deviation weights b_*i*_ within the model. Doing so, a virtual population of 20,000 cases per sex class was generated and used to simulate psoas tendon movement, based on the same provoking kinematics of femoral abduction and rotation.

**FIGURE 2:**
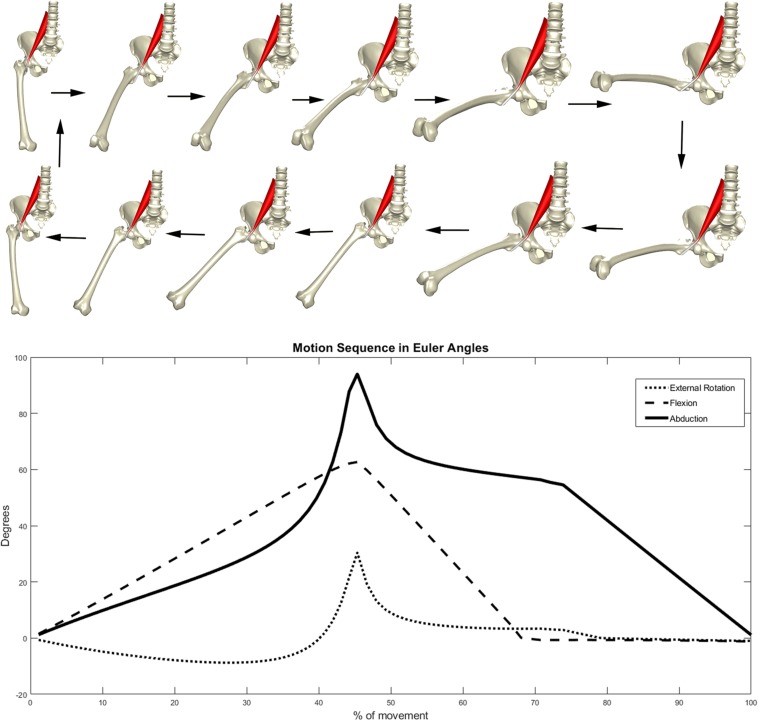
Motion sequence enforced on the virtual models.

**FIGURE 3:**
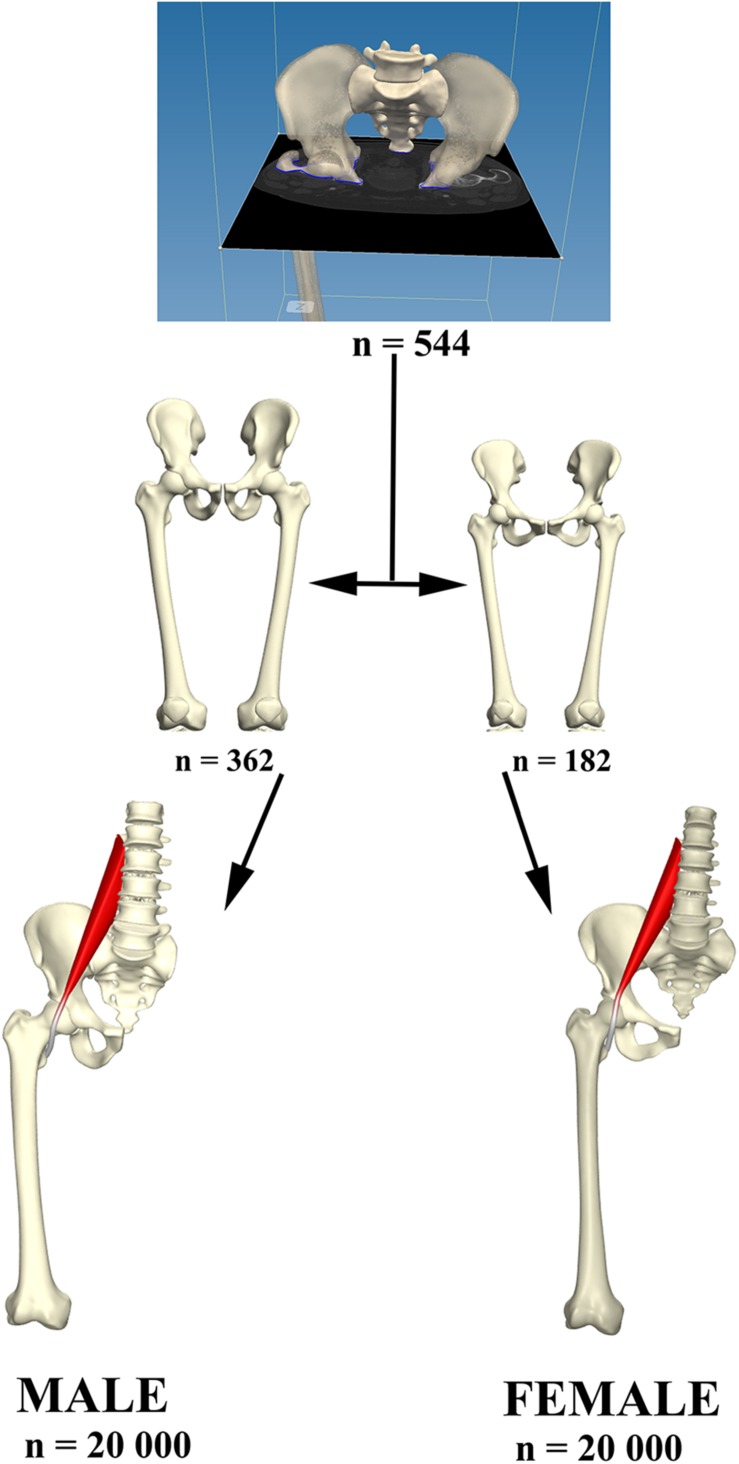
Relevant osteological structures were segmented and use for the construct of a PCA model describing male and female anatomical variance. From each model 20,000 virtual anatomies were created to study psoas tendon behavior in the population created.

Psoas anatomy was predicted and the tendon path modeled according to a previously validated discrete element model of the psoas muscle and tendon (Audenaert et al., 2010, 2019a). Kinematics of circumduction, abduction up to 90 degrees with a concomitant 60 degrees of flexion and 30 degrees of external rotation and returning to the neutral position, were enforced on the virtual population and the position of the psoas tendon was evaluated during that motion performed at an angular velocity of 25 deg/s. Motions were enforced in a stepwise procedure with increasing and decreasing amount of rotation around the x, y, and z axis. The motion sequence that was implemented to describe a circumduction is demonstrated in Figure 2. Decomposition of the rotation matrices was performed to provide an approximative surrogate of clinical semantics in terms of amount of abduction, flexion, and rotation at each step of the motion sequence. The hip joint was modeled as a spherical joint with three degrees of rotational freedom. Psoas tendon positions were evaluated and the angular velocity of tendon movement in relation to the center of the femur head were reported. Simulations were performed on a Dell EMC PowerEdge 940 server hosting 72 cores, processing in parallel at 2.9 GHz. The imposed kinematics were evaluated in 120 consecutive steps. Average simulation time, solving the starting position followed by 120 kinematics steps, was ~3.5 min per case, mounting the required computational cost for 20,000 samples (males and females separately) at 18 h. An overview of the study workflow is presented in Figure 3.

### Statistical Analysis

Given the profound dominance of size in shape models of human anatomy in the Euclidian subspace (Audenaert, 2019), the tendon velocity-shape relationship was evaluated by means of canonical correlation analysis (CCA) in the Mahalanobis subspace. In particular, the PC values of the shape data, serving as predictor variables for the observed peak tendon-velocity measures, were used. Overall explained variance in the observed shape components by the factor peak tendon-velocity was evaluated using partial least squares regression (PLSR). PLSR regression and CCA are highly related to each other and essentially perform the same operation, maximizing the tendon-velocity feature onto the combined shape modes, however, the emphasis is slightly different. In CCA, the goal is to maximize correlation and allows for the statistical interpretation of this observed correlation. In contrast, PLSR maximizes the covariance instead of the correlation, and is typically performed for predictive purposes as a result and again in contrast to CCA. In conclusion, we used CCA to report on the statistical correlation, while PLSR was used to define the predictive value of the peak tendon-velocity feature.

PLSR was further used to describe the male and female consensus shape configurations correlating with low and excess tendon movements. Differences in male and female tendon excursions were further compared between both sexes using the common student t statistics.

## Results

Studying correlations within the virtual cohort based on isolated discrete measures, e.g., femoral anteversion, would obviously brake any important covariations of shape and result in noisy data describing small effects, hence the advantage of using PCA. Nevertheless, discrete measures are valid anatomical descriptions and usually well known by clinical researchers. To be able to translate our findings to the clinical field, a series of commonly used radiographical measures were therefore studied additional to the PCA in the cohort. Findings are described in detail in Tables 1, 2.

**TABLE 1:** Table showing mean values and standard deviations of common used discrete measures to describe femur morphology and sex differences.

	Male (*n* = 20,000)	Female (*n* = 20,000)	*P*-value
Neck shaft angle (°)	125.23 ± 5.48	126.48 ± 5.49	<0.001
Femoral anteversion (°)	9.86 ± 7.02	10.72 ± 6.09	<0.001
Femoral offset (mm)	43.89 ± 6.54	39.11 ± 6.29	<0.001
Ischiofemoral distance (mm)	29.05 ± 4.18	22.23 ± 4.34	<0.001
Head radius (mm)	25.28 ± 1.10	22.37 ± 1.04	<0.001

**TABLE 2:** List of the femur morphological parameters correlations with peak tendon velocity (SSM statistical shape model).

Correlations with peak tendon velocity	Correlation coefficient r Male (*n* = 20,000)	*P*-value	Correlation coefficient r Female (*n* = 20,000)	*P*-value
Neck shaft angle	0.48	<0.001	0.34	<0.001
Femoral anteversion	0.21	<0.001	0.31	<0.001
Femoral offset	−0.54	<0.001	−0.39	<0.001
Ischiofemoral distance	−0.38	<0.001	−0.32	<0.001
Head diameter	−0.14	<0.001	−0.17	<0.001
**Femoral shape (SSM)**	**0.72**	**<0.001**	**0.66**	**<0.001**

**FIGURE 4:**
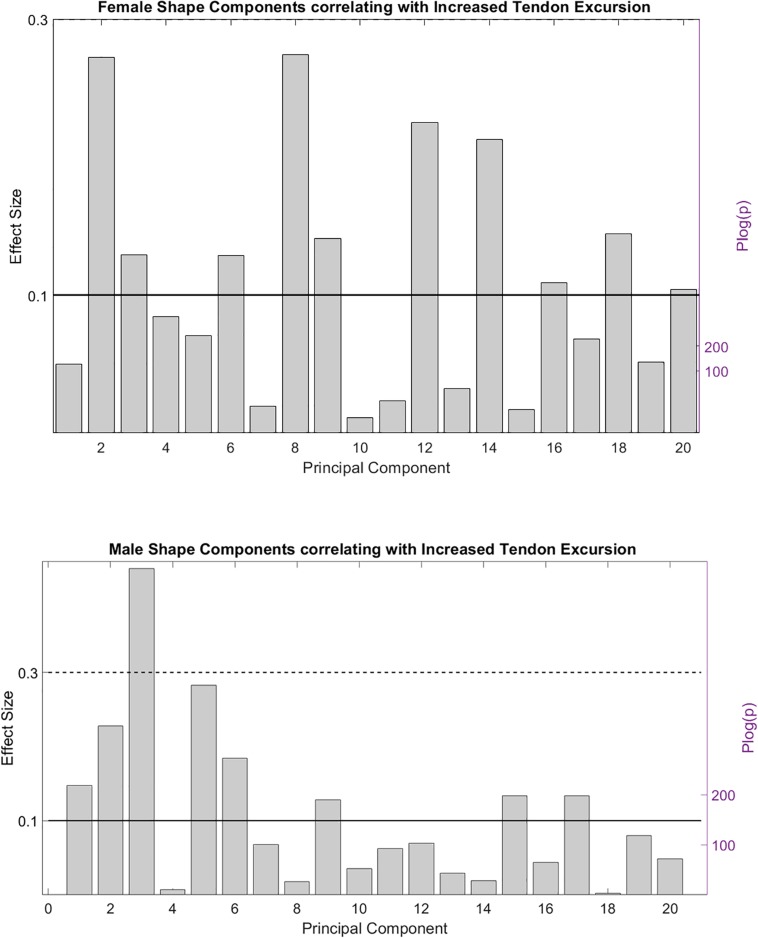
Effect size and statistical significance of the different shape components relating to the observed peak tendon velocity.

PCA is a celebrated linear data compression technique. Although frequently performed, it is absolutely incorrect to interpret isolated PCs as if they were truly existing, independent anatomical variants. PCA can be used anatomically truthful, however, studying observed PC combinations instead of isolated PC findings, with the use of use multivariable statistics. To this end, we firstly evaluated the correlation between the individual shape components and the peak angular velocity of tendon movement. Several contributing PC’s were observed, conferring small to medium effects on peak tendon excursion. Because of the disproportionate size of the study cohort, p values were all small and even converged to zero for some of the variables. Based on the findings it was decided only the first 20 shape components were relevant to further include in the statistical analysis. Effect size and statistical significance of the correlation of these individual shape components are presented in a Manhattan-like plot in Figure 4.

**FIGURE 5:**
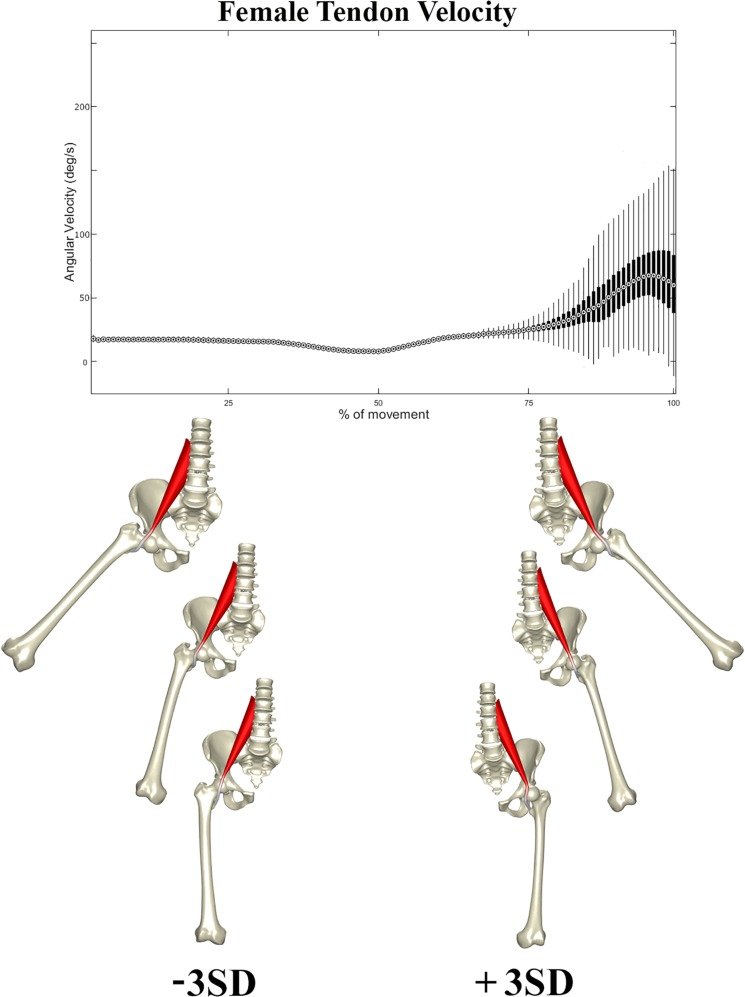
Tendon velocity in the female population studied (Above). Reconstructed shapes correlating with decreased and increased tendon velocity upon return to the neutral position (Below).

**FIGURE 6:**
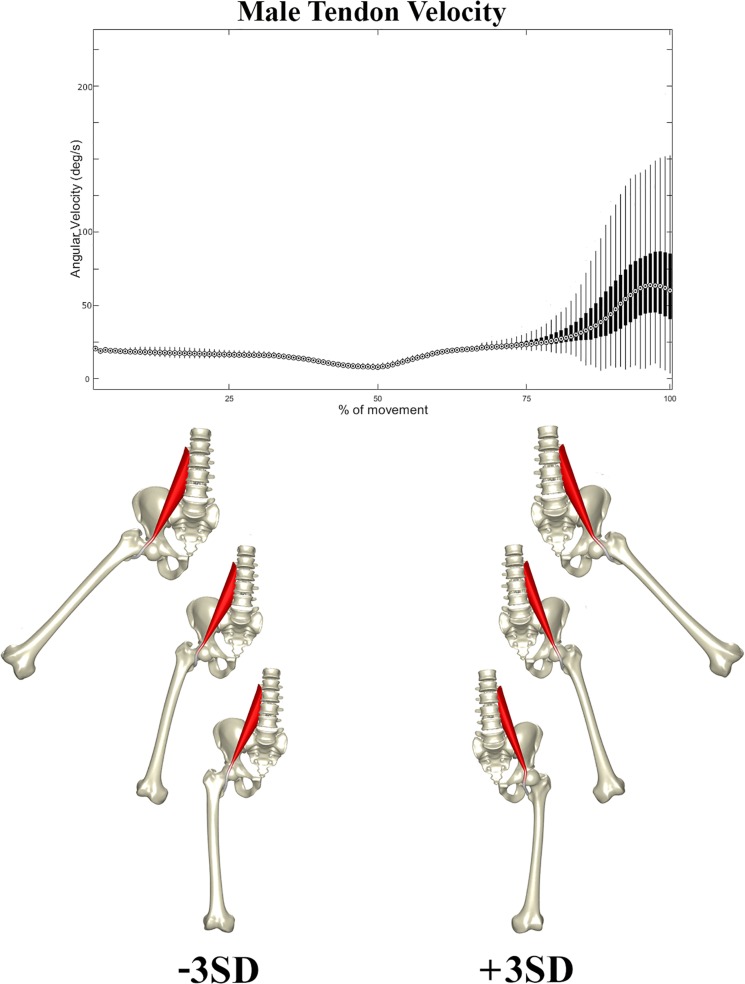
Tendon velocity in the male population studied (Above). Reconstructed shapes correlating with decreased and increased tendon velocity upon return to the neutral position (Below).

In a second stage, the correlation between the composite of shape descriptors and peak tendon velocity was defined by CCA. The observed correlation was considerable in both males (*r* = 0.72, *p* < 1e-300) and females (*r* = 0.66, *p* < 1e-300). The consensus shape configurations relating to the variance in peak tendon velocity were defined using PLSR (Figures 5, 6) images below at ±3 SD. In both populations, abnormal tendon excursion correlated with changes predominantly in the femoral anatomy: increasing femoral head anteversion, valgus, torsion and medial bowing. In essence a rotational variant in femoral shape configuration presenting with a medially located minor trochanter in respect to the pelvis. Interestingly, such shape configuration translates as a decreasing ischiofemoral distance.

A similar pattern of tendon movement was observed in all cases. With ongoing abduction and femoral external rotation, the psoas tendon gradually follows a smooth excursion from the medial portion of the anterior femoral head to lateral, reaching a lateralized position at the valley of the femoral head neck junction (Figures 5, 6).

Upon return to the neutral position, however, the tendon tends to remain hooked in this valley – a stable local minimum in its shortest path position – before initiating its return back medially, causing a sudden increase in tendon velocity at the end of the movement when finding its new optimal medial position over the top of the femoral head. Differences in tendon velocity were observed nearly exclusively in the final stage of the movement, and it was decided to run further statistics against the peak velocity value at this point.

Females were slightly more prone to increased peak tendon movement as compared to males (*p* < 1e-60) The peak angular velocity was found to be slightly higher, 87.5 deg/s in females (range 27.5–247.5 deg/s) as compared to 82.5 deg/s in males (range 19.8–212.5 deg/s).

**FIGURE 7:**
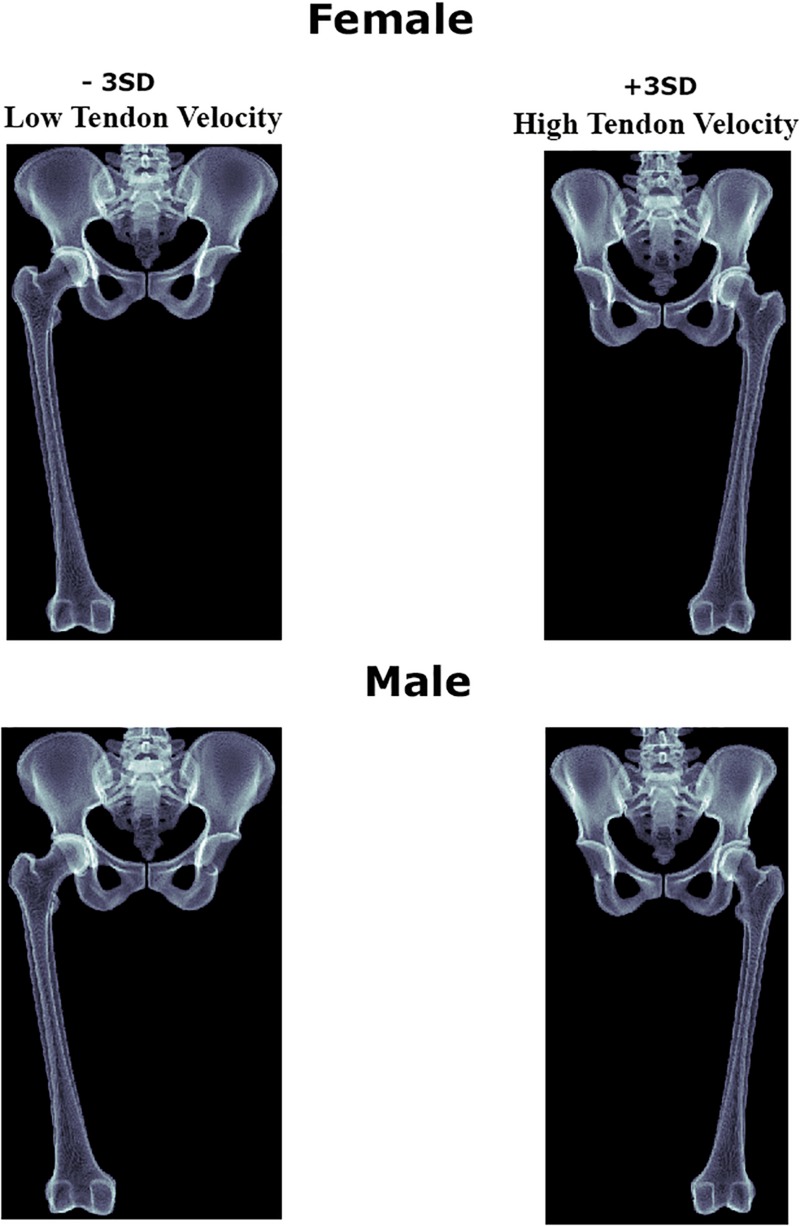
Digitally reconstructed images of the female and male anatomies correlating with decreased and increased psoas tendon excursion upon return to the neutral position.

For the purpose of clinical translation of the findings, digitally reconstructed x-rays from the ±3 SD 3d shapes with low and high tendon velocity were generated and presented in Figure 7.

## Discussion

With larger datasets becoming available, in combinations with increased computational resources, statistical and probabilistic modeling presents an exciting and novel means for non-invasive testing and the evaluation of physiology and biomechanical variability across populations (Bischoff et al., 2014; Gosselin et al., 2014; Sims et al., 2018) In the context of musculoskeletal disease, pathology usually represents an extreme or outlier condition of function, shape, kinetics, or biomechanics at large. The unmet advantage of population numbers in virtual population models allows for the identification and study of these extremes, in cohort sizes beyond what is clinically achievable (Campbell and Petrella, 2016). Based on the current study on a virtual population of 40,000 cases, we were indeed able to describe variation in psoas movement and identify excessive psoas excursion in selected cases.

As mentioned earlier, opinions vary notably on the exact location of psoas tendon movement. Both the femoral head and iliopectineal eminence have been suggested at possible regions of involvement (Howse, 1972). In the present study, the tendon was found to move exclusively laterally and back to the tendon groove. The majority of tendon movement therefore occurred over the femoral head. This finding is in agreement with the sparse *in vivo* dynamic visualization data is available on the topic. Deslandes et al. (2008) observed a similar pattern of motion on dynamic ultrasonography and even claimed absolutely no involvement of the iliopectineal eminence at all. We can, however, based on the particular movement studied, not exclude snapping to occur over the ischiofemoral eminence. In particular during adduction a relative more medial positioning of the minor trochanter might result in such snapping mechanism. Further studies are definitely warranted.

Numerous associated factors, anatomical variants, and pathological conditions, have been reported to increase the risk of psoas movement to become symptomatic snapping. It can be anticipated, that any condition hampering a smooth medial return of the tendon during adduction will further trigger the snapping mechanics. Several clinical studies have indeed described a positive correlation between symptomatic snapping and the presence of labral cysts. The observation of such relationship has not led to any solid etiological proof to date, nevertheless (Blankenbaker et al., 2012). Further dynamic studies are required to elucidate whether snapping becomes symptomatic in the presence of labral pathology or whether labral pathology can occur in case of increased and recurrent psoas tendon movement. Other conditions and structures have also been associated including the iliopsoas bursa, undoubling of the psoas tendon, the ligamentum iliofemoralis, labrum lacerations, paralabral cysts, ligamentum teres lacerations, and instability of the joint (Verhelst et al., 2012; Lee et al., 2013; Philippon et al., 2014; Yen et al., 2015). Moreover, and likely ensuing a similar pathway, snapping has been reported following trauma and surgery of the hip joint (Taher and Power, 2003; Regev et al., 2011; Van Riet et al., 2011; Lee et al., 2013). Similarly, coxa saltans has been reported to occur after total hip prosthesis surgery due to the prosthesis protruding or incorrect positioning of the prosthetic components (Pattyn et al., 2011; Lee et al., 2013; Yen et al., 2015).

A particular interesting observation within this study is the increase in tendon excursion with a decreasing ischiofemoral distance. Ischiofemoral impingement is rather newly described condition with reports describing as well an audible snapping in a significant proportion of the patients reported (Stafford and Villar, 2011). The findings of the presents study seem to associate both snapping hip syndrome and ischiofemoral impingement with a similar underlying torsional femoral dysplasia. In the case of ischiofemoral impingement, excess femoral anteversion has indeed been documented in recent studies (Gomez-Hoyos et al., 2016). This observation might explain the disappointing outcome of psoas lengthening in anteverted femurs in the literature before ischiofemoral impingement was recognized as a distinct clinical condition (Fabricant et al., 2012). Further clinical studies are definitely warranted to elucidate this observed association as to define whether for distinct cases of snapping varisating derotational osteotomy is perhaps more in place than cutting the psoas tendon or removing the lesser trochanter.

The present work needs to be interpreted within is methodological limitations. Firstly, the study remains an *in silico* experiment which will require further *in vivo* confirmation and replication. Secondly, an important restriction of the presented work relates to the population model implemented, namely statistical model of shape obtained from Belgian people and the unknown extent of which findings can be extrapolated to other populations. The complex interaction between gens, environment, and culture, results in a population-based variation. Numerous studies have indeed demonstrated that the appropriate evaluation of this variation necessitates specific standards for each population (Rissech et al., 2013; San-Millan et al., 2017). Nonetheless, in general terms we expect our anatomical model results to be representative for a Western European population.

A second study limitation relates to the motion that was investigated. Snapping has been reported to occur during a broad spectrum of activities, ranging from simple walking to complex ballet exercises. In the present report a circumduction including abduction, flexion and external rotation back to neutral extension and adduction was investigated in order to cover a wide kinematic space. Even though an almost unphysiological 90 degrees of abduction was included in the analysis, increased tendon velocity was only observed in the final part of the motion sequence, close to the return to neutral position. This finding suggests researchers can restrict their analysis in future work in a smaller kinematic range as compared to what we have simulated. Further, this observation suggests more focused research is warranted to investigate common ADL activities in close range to a neutral hip position such as walking and stair climbing. While these activities have been reported to potentially evoke snapping, they mainly involve flexion-extension, with limited abduction and rotation. Considering we investigated a multiplanar hip motion, our findings in terms of the location and mechanism of the snapping can therefore not be generalized to ADL activities that are believed relevant but mainly involve pure flexion-extension. Clearly more work is needed.

In conclusion we found the psoas tendon to exclusively wrap around the anterior femoral head, not conflicting with the iliopectineal eminence. It was associated with female sex, femoral malrotation and a decreased ischiofemoral distance.

## Data Availability Statement

The datasets generated for this study are available on request to the corresponding author.

## Author Contributions

All authors contributed to the study design, data acquisition, and drafting.

## Conflict of Interest

The authors declare that the research was conducted in the absence of any commercial or financial relationships that could be construed as a potential conflict of interest.
